# B7 Family Molecule VSIG4 Regulates Pulmonary Anti-Influenza Immune Responses via C-Type Lectin Signal Pathway

**DOI:** 10.3390/vaccines13101053

**Published:** 2025-10-14

**Authors:** Jianxin Zhu, Dan Lu, Liangyan Zhang, Zhili He, Tianxinyu Ma, Yakun Sun, Wenjing Yu, Xiaolan Yang, Yeqing Tu, Yitai Fang, Deyu Li, Rui Zheng, Tao Li, Jin Zhao, Hui Wang

**Affiliations:** 1 School of Basic Medical Sciences, Anhui Medical University, Hefei 230032, China; 17719530652@163.com (J.Z.); 17782307969@163.com (T.M.); goyuii@163.com (Y.F.); viollet2017@126.com (R.Z.); 2 State Key Laboratory of Pathogen and Biosecurity, Academy of Military Medical Sciences, Beijing 100071, China; 18807007914@163.com (D.L.); polini@live.cn (L.Z.); lili892017173@163.com (Z.H.); sykddyx@163.com (Y.S.); cpu_ywj@163.com (W.Y.); yangxiaolanxl@163.com (X.Y.); tyq2962280944@163.com (Y.T.); 18842345409@163.com (D.L.); litaobmi@126.com (T.L.)

**Keywords:** VSIG4, B7, influenza, T cell

## Abstract

**Background**: As the member of the B7 family, V-set and immunoglobulin domain-containing 4 (VSIG4) plays an essential role in regulating immune responses against bacterial infection, autoimmune disease, and chronic viral infection. However, the role of VSIG4 in acute viral infections remains largely unclear. **Methods**: Here, we constructed a gene-targeted VSIG4-deficient mouse model and then infected it with influenza to explore the detailed VSIG4-involved mechanism. **Results**: Our results demonstrated that the gene-deficient mice exhibited reduced survival rates, ranging from 25% to 50%, after being infected with different influenza virus strains. At the sites of infection, an increased number of CD8^+^ T cells, along with heightened expression of pro-inflammatory cytokines, e.g., Il-6 and TNFα, may have contributed to tissue damage. The recombinant VSIG4 protein slightly improved protection from the influenza challenge, suggesting regulatory functions of VSIG4 during infection. Using in vitro cell models, we show that the type C lectin receptor pathway member DC-SIGNR1 (CD209) is an essential factor during acute virus infection. The affinity and CO-IP tests indicated an interaction between CD209 and VSIG4, but not through protein modification. **Conclusions**: Therefore, VSIG4 functionally protected mice by regulating the type C lectin receptor pathway to inhibit excessive Th1 immune responses and inflammation. Our findings highlight the importance of considering immune homeostasis in the development of therapies for severe infections.

## 1. Introduction

As a member of the B7 family, V-set and immunoglobulin domain-containing 4 (VSIG4) plays a critical role in the regulation of immune responses [1]. VSIG4 is selectively expressed in resting tissue-resident macrophages and serves as a key marker that facilitates the clearance of bacterial pathogens by binding to complement components C3b or inactivated C3b (iC3b) [2]. Moreover, VSIG4 plays a role in suppressing macrophage-driven inflammation by transcriptionally inhibiting Nlrp3 and IL-1β expression in the context of inflammatory diseases such as multiple sclerosis (MS) and autoimmune arthritis [3]. Studies using animal models of autoimmune type 1 diabetes have demonstrated that VSIG4 inhibits T cell proliferation and facilitates the differentiation of Foxp3^+^ regulatory T cells (Tregs) through interaction with an unidentified receptor on T cells [4]. Currently, most research on macrophages focuses on tissue damage and disease onset induced by tissue-specific chronic excessive inflammation [5,6,7]. As an important marker of tissue-resident populations, the function and mechanism of VISG4 in these disease models have been described [8,9]. However, the potential role of VSIG4 in controlling acute viral infection, as well as its underlying mechanisms, has yet to be elucidated.

Under infected or autoimmune disease conditions, macrophages sense environmental stimuli and subsequently induce primary T cell response to defend against pathogens or induce tissue damage [10]. Thus, maintaining immune balance has become the predominant topic in infection, cancer, and autoimmune disease. Macrophages may modulate T cell responses through multiple mechanisms. The most important mechanism is that T cells are regulated by some stimulated molecules secreted by macrophages in tissues. Macrophages, especially tissue-resident macrophages, can express some coinhibitory molecules of the CD28 and TNFR families. These molecules have been proven functional in regulating T cells or T-reg cells in a few studies, such as PD-1 and TIM-4 [11,12]. However, the influence of the tissue microenvironment and immune checkpoints on T cell responses remains inadequately understood.

The influenza virus represents a leading cause of severe viral respiratory illness, and the secondary bacterial infections that frequently follow can result in substantial morbidity and mortality, impacting 3 to 5 million individuals each year [13]. Understanding the mechanisms by which VSIG4 mediates this acute viral infection and how VSIG4 facilitates respiratory immunity is of growing interest. Here, we used an influenza virus-infected VSIG4-deficient animal model to define the detailed mechanisms involved in acute virus infection. The study demonstrated that VSIG4 deficiency heightens the vulnerability of pulmonary immunity to the influenza virus. This heightened susceptibility is associated with elevated numbers of CD8^+^ T cells and excessive production of pro-inflammatory cytokines, ultimately leading to tissue damage. DC-SIGNR1 (CD209), a member of the type C lectin receptor family, interacts with VSIG4 to suppress excessive Th1 immune responses. The defects observed in the mouse models employed in this study provide strong evidence that VSIG4 is essential for maintaining innate immune homeostasis in defense against pulmonary infection.

## 2. Materials and Methods

### 2.1. Mice

All animal studies were conducted in accordance with Beijing Institute of Microbiology and Epidemiology Animal Care and Use Committee guidelines. VSIG4 knockout mice on a C57BL/6J background were generated by our Laboratory Animal Center using the CRISPR/Cas9 gene editing technique to delete a 624 bp fragment in exon 2. The primers used for verification were as follows: Forward, 5′-cacacaa-tagctagcacaaccagtg-3′; Reverse, 5′-aaattgggagatgtacttggtggga-3′. Ten mice heterozygous for the VSIG4 gene (VSIG4^+^/^−^, Heter) and ten homozygous knockout mice (VSIG4^−^/^−^, KO) were randomly selected. The mice were acclimated for one week under grouped housing conditions. Fresh fecal samples (3–6 pellets per mouse) were collected into sterile microcentrifuge tubes, labeled, immediately frozen in liquid nitrogen, and stored at −80 °C for subsequent analysis. C57BL/6 wild-type mice (5-week-old, weighing 14–16 g) were obtained from Weitonglihua Company, Beijing, China. All experimental mice were bred in a specific pathogen-free facility at our institute. The experimental mice were matched for age and sex and cared for according to the guidelines of the institute (IACUC-IME-2024-030).

### 2.2. Virus Infections

Influenza A virus (IAV) strains A/Puerto Rico/8/1934 (PR8), A/Vietnam/1194/2004 (H5N1) and A/Beijing/501/2009 (BJ501) were propagated in 10-day-old specific pathogen-free chicken embryos. The mice were infected intranasally with 10^3.5^ TCID_50_ influenza PR8, 10^3.5^ TCID_50_ H5N1, and 10^5.5^ TCID_50_ BJ501. They were monitored and weighed at least once daily after infection. The mice that became recumbent or lost more than 30% of their body weight were considered moribund and were euthanized. When indicated, the wild-type mice were challenged intranasally with 10^3.5^ TCID_50_ influenza PR8 and then supplied with 10 μg VSIG4 protein in vivo by i.v. injection (SinoBiological, Beijing, China) every day; the control mice received injections of PBS. All mice were observed daily and harvested on day 8 post-infection. The viral titers in the lungs were evaluated by qRT-PCR to test the influenza gene M1 expression (M1-Forward primer: AAGACCAATCCTGTCACCTCTG; M1-Reverse primer: CAAAACGTCTACGCTGCAGTCC), and the levels of inflammatory cytokines and chemokines in whole lung tissue were determined by Luminex assay.

### 2.3. Flow Cytometry

Preparation of lung single cells is described below. In brief, cells isolated from lung tissues were ground with glass rods and incubated with Fc Block (clone 2.4G2) for 15 min at 4 °C, then washed 3 times. For enumeration of CD45 cells, macrophages, and neutrophils, pulmonary lymphocytes were stained on ice with anti-CD45 (clone RM-5), anti-CD11b (clone M1/70), and anti-Ly6G (clone 1A8), anti-Gr-1 (clone RB6-8C5), anti-CD3 (clone 17A2), anti-CD4 (clone RM4-5), anti-CD8a (clone 53-6.7), and anti-VSIG4 (eBioscience, San Diego, CA, USA). The gating strategies for T cells range from lymphocytes to single cells, to live cells, to CD45+ cells, to CD3e+/CD4+/CD8+. The gating strategies for macrophages are from lymphocytes to single cells, to live cells, to CD45+ cells, to F4/80+/CD11b+. Data were gated for forward scatter/side scatter, collected on an FACSCanto II (BD Biosciences, San Jose, CA, USA), and analyzed using Flow Jo software 10.0 (Tree Star, Ashland, OR, USA). Flow cytometry data were visualized using cloud plots, and the frequencies of T cells and macrophages were quantified with Flow Jo software 10.0. Statistical differences among the groups were analyzed by one-way ANOVA.

### 2.4. Luminex Multiplex Cytokine Test

The levels of 34 cytokine molecules were measured using a Luminex-based bead array, the ProcartaPlex Hu Cytokine/Chemokine Panel 34 plex (Invitrogen, Thermo Fisher Scientific, Wilmington, DE, USA), for the following biomarkers: IL-18, IL-6, IL-2, G-CSF, IL-12p70, IL-13, IL-10, IFN-γ, TNF-α, IL-1β, and so on. In brief, 25 µL Broncho alveolar lavage fluid (BALF) and serum samples were analyzed for cytokine levels using a multiplex immunoassay with fluorescent-labeled beads conjugated to specific monoclonal antibodies targeting the molecules of interest, following the manufacturer’s instructions [14]. Appropriate standards, including 34 beads and quality control (provided by the manufacturers with the test sample plate), were used. The results were interpreted from the standards using the Luminex 200 system. The software determined and interpreted the data and concentrations of analytes in the samples based on the standards run in the test.

### 2.5. Transcriptome Sequencing

**Scheme 1:**
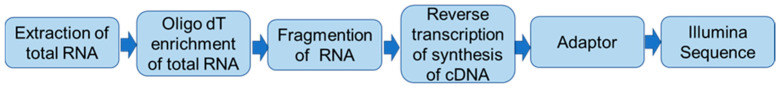
Workflow of transcriptome sequencing.

Total RNA from the lungs of VSIG4^−/−^ and wild-type mice was isolated using RNAiso plus reagent (Takara, Dalian, China) in accordance with the manufacturer’s instructions. DNase I digestion was used to eliminate genomic DNA contamination. The quality and purity of RNA was checked using a Nanodrop ND-1000 spectrophotometer (Thermo Fisher Scientific, Wilmington, DE, USA). A total of 1 μg RNA per sample was sent to Majorbio (Shanghai, China) for RNA sample preparation, sequence mapping, assembly, and gene functional annotation. The detailed workflow of transcriptome sequencing is shown in Scheme 1.

### 2.6. Construction of Recombinant Adenovirus Encoding mVSIG4

The recombinant adenovirus vector encoding VSIG4 was generated using the Adeno-X™ Expression System (Clontech, Palo Alto, CA, USA) following the manufacturer’s instructions. Briefly, the mVSIG4 cDNA was cloned into the shuttle vector FV012 (pCDNA3.1) containing EGFP. The desired replication-deficient adenovirus containing the full-length cDNA of mVSIG4 was generated by homologous recombination through co-transfection of plasmids pCDNA3.1-mVSIG4 and pBHG1oXE1 and 3Cre in HEK 293 cells using the DOTAP liposome reagent (Roche, Mannheim, Germany). After several rounds of plaque purification, the adenovirus containing the GFP-mVSIG4-3*flag (Ad-GFP-mVSIG4-3*flag) gene was amplified and purified from the cell lysates by banding twice in CsCl density gradients. Viral products were desalted and stored at −80 °C in PBS containing 10% glycerol (*v*/*v*). A second recombinant El- and E3-deleted adenovirus carrying the LacZ protein under the control of the CMV promoter (rAd-LacZ) was used as a control vector for DC transduction.

### 2.7. Cellular Infection

The day before infection, approximately 1 × 10^6^ of RAW264.7 cells (mouse colon macrophage) were seeded in DMEM (10% fetal bovine serum, Gibco, Grand Island, NE, USA) on six-well plates or coverslips without antibiotics. VSIG4 recombinant protein (SinoBiological, Beijing, China) was added to fresh DMEM medium at a 5 μg/mL concentration, and the recombinant Fc protein (SinoBiological, Beijing, China) served as a control. Meanwhile, the cells were infected with PR8 at a multiplicity of infection (MOI) = 0.01. Phosphate-buffered saline (PBS)-treated cells were used as controls. The supernatants and cells were collected for viral burden, levels of inflammatory cytokines, and chemokines. Ad-GFP-mVSIG4-3*flag virus was added to the wells at a series of MOIs—10, 20, 30, and 40—and the RAW264.7 cells were harvested after 24 h of incubation.

### 2.8. Immunoprecipitation and Immunoblotting Assay

Peritoneal macrophages (PETs) were isolated from the mouse peritoneal cavity 4 days after thioglycolate (Sigma, St. Louis, MO, USA) injection. The macrophages were cultured in DMEM supplemented with 10% (*v*/*v*) fetal bovine serum (FBS) and penicillin–streptomycin (100 U/mL). For the immunoprecipitation assays, PETs were collected after 48 h and then lysed in lysis buffer (25 mM Tris∙HCl pH 7.4, 150 mM NaCl, 1%Nonidet P-40, 1 mM EDTA, 5% glycerol) supplemented with protease inhibitor mixture (Thermo Fisher Scientific) on ice for 30 min with periodic mixing. The whole cell lysates were centrifuged and then incubated with an anti-Flag (Sigma) or anti-mVSIG4Ab overnight at 4 °C, followed by further incubation with protein A beads (Sigma) for 2 h at 4 °C. The cell lysates were removed from the refrigerator and thawed on ice. The sample detection mixture was prepared. Then, following the manufacturer’s instructions, the primary antibody dilution for the target protein, secondary antibody, and chemiluminescent substrate were sequentially prepared. The prepared reagents were sequentially added to the detection plate. The plate was covered and centrifuged at 25,000 rpm at room temperature, ensuring that it was properly balanced. The detection plate was placed into the new instrument (Protein sample, WesTM WS-3493), the capillaries were retrieved and loaded into the automated Western blot analyzer (ProteinSimple, San Jose, CA, USA), the computer was connected to it, the parameters were set, and the program was started. Upon completion of the program, protein expression levels were analyzed using Compass for SW software 5.0. Primary antibodies used included anti-CD209 (Biolegend,147802), anti-Ac, anti-phospho-Ser/Thr, anti-phospho-Tyr, anti-Ub, anti-VSIG4 (Abcam, Cambridge, UK, ab252933), anti-BCL3, anti-IKKE, anti-LSP1, and anti-CYLD (1:1000; ABclonal, Wuhan, China). After three washes with TBS containing 0.1% Triton X-100, goat anti-rabbit or anti-mouse HRP-conjugated secondary Ab (1:5000; Cell Signaling Technology, Danvers, MA, USA) was incubated for 1 h at room temperature. The final detection of protein was performed using Compass for SW software 5.0.

### 2.9. Confocal Immunofluorescence Microscopy

PEMs were fixed with 4% paraformaldehyde at room temperature for 15 min, permeabilized with PBS containing 10% FBS, 3% BSA, and 0.5% Triton X-100 for 15 min, and then incubated with diluted primary Abs overnight at 4 °C. After two washes with PBS, the cells were incubated with secondary Abs conjugated to Alexa Flour^®^ 488 and Alexa Flour^®^ 555 (Abcam) at room temperature for 1 h. Cell nuclei were stained with DAPI (Roche) for 10 min, followed by three PBS washes. Images were captured using fluorescence microscopy (Zeiss LSM880, Carl Zeiss AG, Oberkochen, Germany).

### 2.10. Histology

Tissues were fixed in 10% neutral buffered formalin, embedded in paraffin, sectioned, and stained with hematoxylin and eosin (H&E). Pathological foci in each section—defined as areas with extensive inflammatory cell infiltration accompanied by submucosal edema—were evaluated. Representative photomicrographs were captured at ×100 magnification.

### 2.11. Statistical Analysis

Statistical analyses were performed using the program Prism 8.0 (GraphPad Software, Inc., La Jolla, CA, USA). Values are expressed as mean ± SD. Data were analyzed by unpaired Student’s *t*-test (normal distribution) or one-way ANOVA, followed by Dunnett’s multiple comparison tests. Survival data were analyzed by log rank tests, and *p* < 0.05 was considered to be statistically significant.

## 3. Results

### 3.1. VSIG4 Gene Deficiency Increased Susceptibility to Influenza Virus Infection in Mice

**Figure 1:**
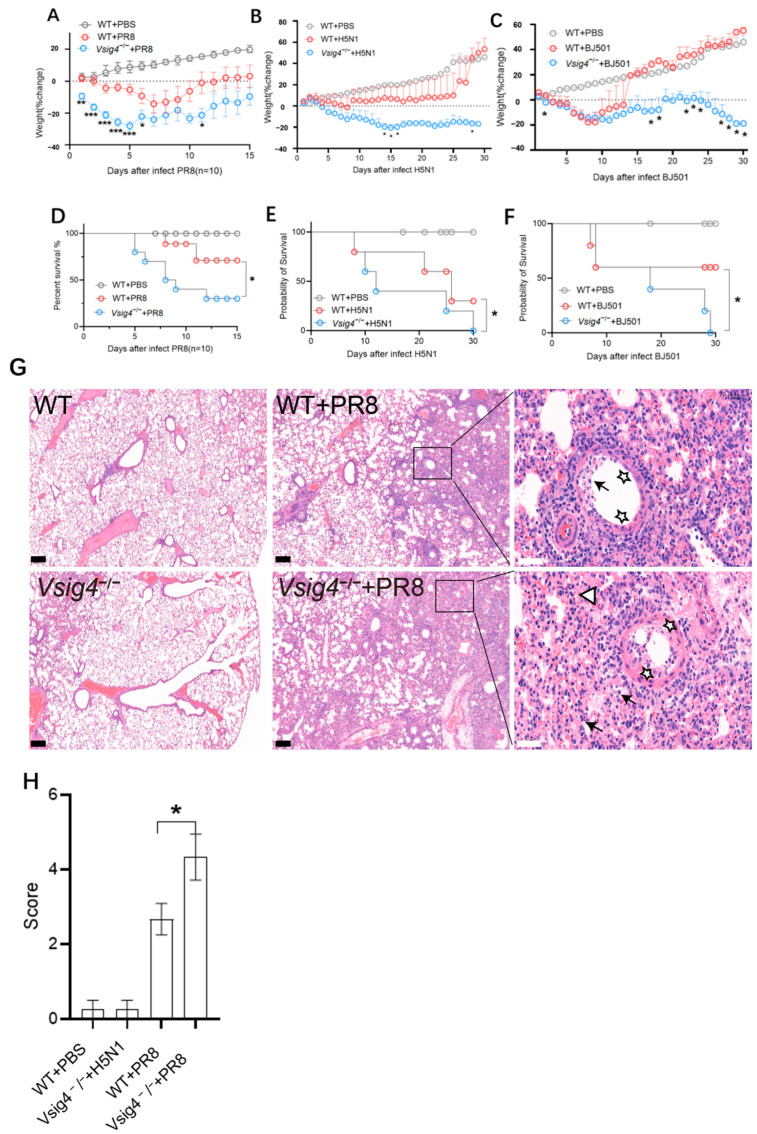
VSIG4 gene deficiency influences susceptibility to influenza virus infection in the lung. VSIG4 gene-deficient mice and control mice were inoculated with a sublethal dose (10^3.5^ TCID_50_) of influenza PR8 (**A**,**D**), A/Vietnam/1194/2004 (H5N1) (10^3.5^ TCID_50_) (**B**,**E**), and A/Beijing/501/2009 (10^5.5^ TCID_50_) (BJ501) (**C**,**F**), and we monitored survival and weighed mice every day. On day 8 after the inoculations, the mice were euthanized and lung pathology was evaluated. The pentagram indicates epithelial degeneration and necrosis, the arrow points to inflammatory cell infiltration and foamy cells, and the triangle indicates inflammatory exudate (**G**,**H**). All error bars are SEM. * *p* < 0.05, ** *p* < 0.01, *** *p* < 0.001.

Before our study, we observed and tested the condition of VSIG4 gene-deficient mice and found that both survival (Supplementary Materials Figure S1A,B) and some physiological indexes (Supplementary Materials Figure S1C) were similar to those of wild-type mice. To investigate the role of VSIG4 during acute viral infection, VSIG4-deficient mice were intranasally challenged with a sublethal dose (10^3.5^ TCID_50_) of influenza A virus (A/Puerto Rico/8/1934, PR8). We found that VSIG4 deficiency alone led to mortality and significant body weight loss in the mice (Figure 1A,D). The control mice readily survived the sublethal intranasal dose challenge with influenza virus. Parallel evaluations of body weight changes over the course of infection suggested that the control mice experienced less severe disease than did the VSIG4 gene-deficient mice. This susceptibility did not correlate with increased virus titers in the lung, because we did not find any difference in viral load between the VSIG4 gene-deficient and wild-type mice. Meanwhile, the VSIG4-deficient mice were also challenged intranasally with sublethal doses of influenza A virus strains A/Vietnam/1194/2004 (H5N1, 10^3.5^ TCID_50_) (Figure 1B,E) and A/Beijing/501/2009 (BJ501, 10^5.5^ TCID_50_) (Figure 1C,F) to assess their susceptibility. We observed that the VSIG4-deficient mice succumbed to sublethal infection, regardless of the influenza virus strain used. To further evaluate the protective role of VSIG4 during influenza infection, lung tissue damage was assessed. On day 8, post-inoculation with a sublethal dose of the PR8 virus, the lungs of the VSIG4-deficient mice exhibited severe pneumonia. Bronchial epithelium at all levels exhibited severe degeneration and necrosis, accompanied by hemorrhage and edema around the blood vessels. Large numbers of inflammatory cells and an inflammatory exudate infiltrated the areas surrounding the alveoli. In contrast, the control mice showed moderate pneumonia. Bronchial epithelium at all levels showed degeneration and necrosis, with infiltration of inflammatory cells. Foam cells were visible in some fields of view (Figure 1G). Pathological scoring (Figure 1H) confirmed significant differences in both the number and severity of pulmonary lesions characterized by marked edema. These findings demonstrate that susceptibility to influenza virus infection is strongly associated with VSIG4 deficiency.

### 3.2. VSIG4 Gene Deficiency Upregulated Expression of Inflammatory Factors

**Figure 2:**
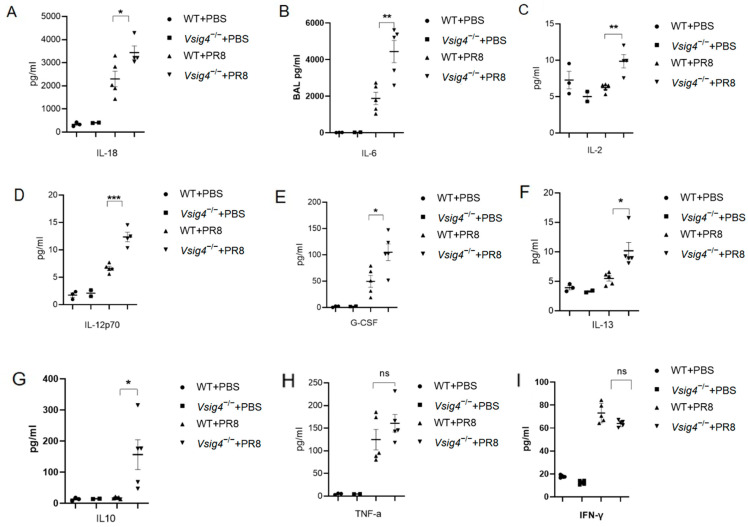
VSIG4 gene-deficient mice and control mice were inoculated with a sublethal dose of influenza PR8, and tissue samples from their lungs were harvested on day 8. BALF levels of IL-18 (**A**), IL-6 (**B**), IL-2 (**C**), IL-12p70 (**D**), G-CSF (**E**), IL-13 (**F**), IL-10 (**G**), TNF-α (**H**), and IFN-γ (**I**) were measured (*n* = 8–10 per group). All data are shown with real concentration, but normalized with log 10 for the one-way ANOVA statistical test. All error bars are SEM. * *p* < 0.05, ** *p* < 0.01, *** *p* < 0.001.

Since macrophages are closely associated with chronic excessive inflammation, we should evaluate pro-inflammatory responses in the lung. We measured inflammation markers in the lungs of the mice on day 8 after inoculation with a sublethal dose of influenza virus PR8. Consistent with our previous discoveries of lung tissue damage (Figure 1G), high levels of markers of inflammation were observed in the VSIG4 gene-deficient mice, including levels of IL-18 (Figure 2A), IL-6 (Figure 2B), IL-2 (Figure 2C), IL-12p70 (Figure 2D), G-CSF (Figure 2E), IL-13 (Figure 2F), and IL-10 (Figure 2G) in BALF. There were no differences between the two groups in the other 27 cytokines and chemokines, including TNFα (Figure 2H) and IFNγ (Figure 2I). To further investigate the impact of VSIG4 molecular-mediated regulation of pro-inflammatory responses during influenza virus infection, the serum was used to evaluate the inflammatory secretion. We found that the levels of serum IL-6 (Supplementary Materials Figure S2A) and serum IL-1β (Supplementary Materials Figure S2B) had increased in the gene-deficient mice. Thus, as an important marker of tissue-resident macrophages, VSIG4 molecules played an important role in modulating pro-inflammatory responses in these infected animal models.

### 3.3. CD8^+^ T Cell and Macrophages Were Involved in Pulmonary Anti-Influenza Immune Responses

**Figure 3:**
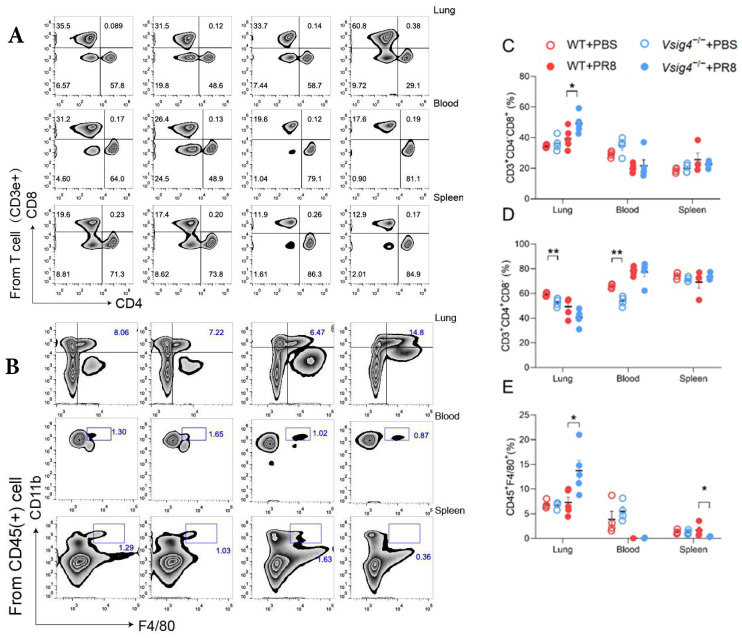
VSIG4 gene deficiency altered immune cell responses. On day 8 post-inoculation with a sublethal dose of PR8 virus, the lungs of the VSIG4-deficient mice were harvested. Immune cell counts, including CD8^+^ T cells (**A**,**C**) CD4^+^ T cells (**A**,**D**), and macrophages (**B**,**E**), were evaluated by flow cytometry and expressed as percentages of immune cells (mean ± SEM). * *p* < 0.05, ** *p* < 0.01.

To further investigate the mechanism of VSIG4-mediated protection from influenza virus infection, we evaluated the immune responses against infection. We evaluated immune cell counts in different organs after inoculating the mice with the PR8 influenza virus. We observed that the number of pulmonary CD8^+^ T cells (Figure 3A,C) and macrophages (M) (Figure 3B,E) increased significantly in the VSIG4 gene-deficient mice. The number of pulmonary CD4^+^ T cells did not change in the gene-deficient mice after infection (Figure 3A,D). CD8^+^ T cells play a major role in mediating protection against influenza strains [15]. However, excessive cellular immune responses to influenza may exacerbate susceptibility to respiratory infectious diseases [16]. The depletion of CD8^+^ T cells impaired protective immunity against influenza virus infection [17]. Therefore, maintaining appropriate levels of T cell responses is crucial during viral infection. CD8^+^ T cells and macrophages are involved in regulating pro-inflammatory responses or identified as main cell lines that secrete inflammatory factors. Just like in tumor mouse models, our results suggest that the abundance of CD8^+^ T cells and macrophages is important for regulating pro-inflammatory responses during acute virus infections, which induce tissue damage. Since VSIG4 molecular influences immune protective function during sublethal dose influenza virus infection, it is worth exploring the mechanism during acute viral infection.

### 3.4. VSIG4 Regulated Anti-Virus Immune Responses Through Type C Lectin Receptor Pathway

**Figure 4:**
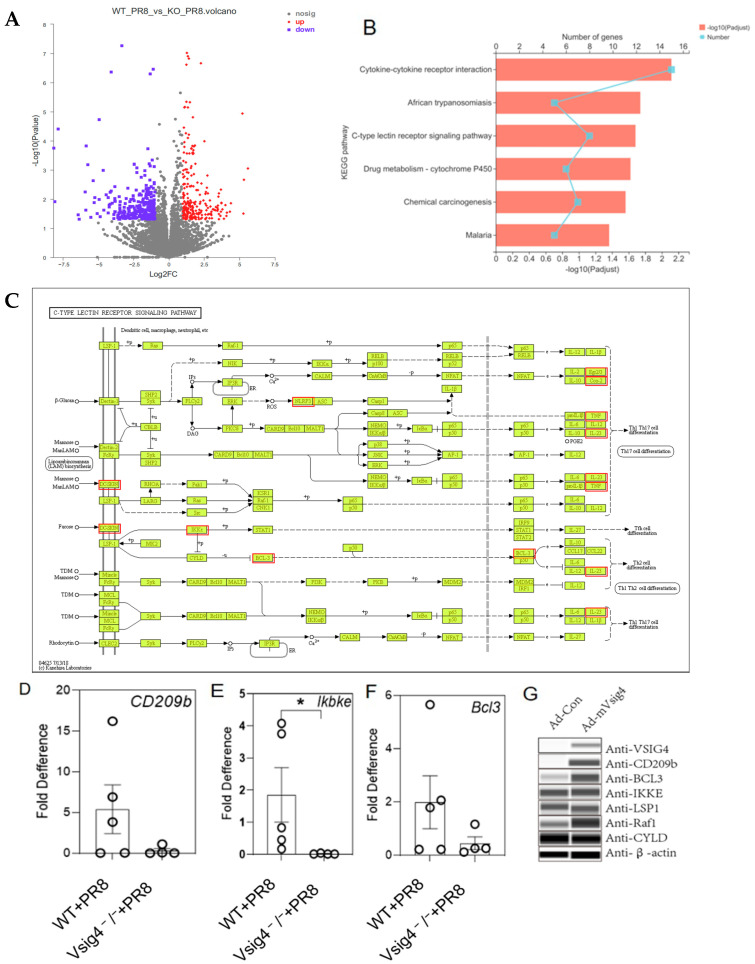
VSIG4 is essential in the type C lectin receptor pathway. Transcriptome sequencing technology was used to evaluate the gene expression during influenza virus infection. (**A**) In the lungs of VSIG4^−/−^ mice, 230 genes were upregulated and 298 genes were downregulated. (**B**) Pathway enrichment analysis. (**C**) C-type lection receptor signaling pathway analysis, with the regulated factors shown with fold differences (mean ± SEM). CD209 (**D**), IKKE (**E**), BCL3 (**F**). (**G**) The regulated factors of the C-type lection receptor signaling pathway were evaluated with Western blot; * *p* < 0.05.

To investigate the mechanism in this model, transcriptome sequencing technology was used. The VSIG4 gene-deficient and control mice were infected with influenza virus, and then the lung tissues were harvested for the next step. The total RNA was extracted and sent to the company for transcriptome sequencing and analysis. To identify differentially expressed genes, unigenes from the lungs of VSIG4^−/−^ and wild-type mice were compared. In the lungs of VSIG4^−/−^ mice, 230 genes were upregulated and 298 genes were downregulated (Figure 4A). In order to analyze whether DEGs were overrepresented in specific pathways, pathway enrichment analysis was performed (Figure 4B). The top 3 enriched pathways were “cytokine-cytokine receptor interaction”, “African trypanosomiasis”, and “C-type lectin receptor signaling pathway”. Because the factors of the C-type lectin receptor signaling pathway upstream and downstream were both changed in this animal model, the C-type lectin receptor signaling pathway was analyzed (Figure 4C). The results showed that VSIG4 gene deficiency upregulated pro-inflammatory genes, e.g., Tnf, il-23a, and Nlrp3, and downregulated pro-inflammatory genes, e.g., Ptgs2, Egr3, Ikke, Bcl3, and CD209b. The mRNA expression validation analysis of related genes found that the type C lectin receptor pathway DC-SIGNR1 (CD209)-IKKe-BCL3 gene group was closely related to VSIG4. To further investigate the mechanism involved in the process, the overexpressing VSIG4 RAW264.7 cell model was constructed according to the instructions. Using this cell model, we found that some factors in the C-type lectin receptor signaling pathway were unregulated, e.g., CD209 (Figure 4D), BCL3 (Figure 4E), IKKE (Figure 4F), LSP1, and Raf1, especially CD209 (Figure 4G).

### 3.5. Interaction of VSIG4 and CD209 in Macrophages Regulates Pro-Inflammatory Responses

**Figure 5:**
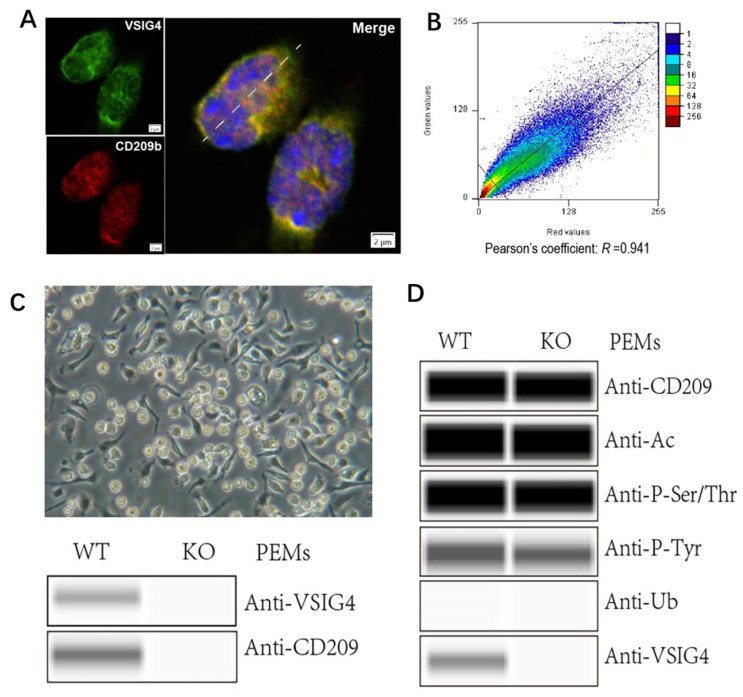
Interaction of VSIG4 and CD209. Peritoneal macrophages (PETs) were obtained from the mouse peritoneal cavity 4 d after thioglycolate injection, and then assayed by different methods. Immunofluorescence showed cellular colocalization of VSIG4 and CD209 (**A**). Immunofluorescence co-localization micrographs: green channel, red channel, and merged composite channel. Pseudocolor, wherein the green signal represents VSIG4 protein, the red signal represents CD209b protein, and the blue signal represents the nucleus. The cell membrane signal is not displayed. White scale bar: 2 μm. Correlation analysis of red and green signal values was performed along the white dashed line in the merged composite channel image (**B**). The x- and y-axes represent the red and green signal values, respectively. Signal distribution density is indicated by the density scale in the upper left corner. The black line represents the trend of the signal value distribution. The immunoprecipitation assays showed that the interaction between VSIG4 and CD209 was not impaired in the protein modification level (**C**,**D**).

At the outset of exploring VSIG4’s role in immune responses, we hypothesized that VSIG4 might be involved in CD209-mediated signaling. Subsequently, we investigated the potential interaction between VSIG4 and CD209. A protein binding affinity analysis performed using the biacore revealed that the VSIG4 protein did not bind to CD209. Then, the tissue-resident macrophages in the abdominal cavity of the VSIG4 gene-deficient mice and control mice were isolated. Confocal microscopy revealed a potent interaction between VSIG4 and CD209. In this study, immunofluorescence showed cellular colocalization of VSIG4 and CD209 (Figure 5A). The Pearson correlation coefficient of this two-protein distribution is over 0.9, which indicates a strong correlation between VSIG4 and CD209 (Figure 5B). The interaction between VSIG4 and CD209 has been previously reported; however, the exact mechanism of this interaction remains unclear. To investigate the regulation of CD209 within the C-type lectin receptor signaling pathway, we evaluated CD209 protein modifications. Both overexpression and endogenous immunoprecipitation assays demonstrated that the interaction between VSIG4 and CD209 was not affected by protein modifications (Figure 5C,D), suggesting that VSIG4 may interact with activated CD209 through alternative mechanisms. The detailed mechanism will be explored in the near future. These findings indicate that VSIG4 participates in activating the C-type lectin receptor signaling pathway in an unknown way, thereby regulating T cell maintenance and pro-inflammatory responses during influenza virus infection.

## 4. Discussion

While inflammatory responses are essential for host defense against pathogens, uncontrolled expression of pro-inflammatory cytokines may cause tissue damage [18]. The identification and characterization of molecules and pathways that modulate inflammation and eliminate excessive inflammatory cells are therefore important in preventing acute inflammation-induced tissue damage [19].

In our study, the VSIG4^−/−^ mice became more susceptible to influenza virus infection. The control mice readily survived sublethal dose intranasal challenge with influenza virus. Meanwhile, we observed elevated expression of pro-inflammatory cytokines and prominent inflammatory foci with varying degrees of lung damage in the VSIG4-deficient mice following influenza infection. The increased proliferation of CD8^+^ T cells and tissue-resident memory T cells was associated with heightened inflammation. Our findings demonstrate that VSIG4, a B7 family-associated protein specifically expressed in resting macrophages, plays a regulatory role in this process [1]. However, the potential clinical treatment with modulating factors involved in the inflammatory signal pathway has become a much-debated topic in recent years. These results indicate that modulation of VSIG4 signaling may offer a promising therapeutic strategy for managing inflammation during acute viral infections.

Based on our observation, one useful way to manipulate the pro-inflammatory cytokine expression is by supplying VSIG4 recombinant protein. We purchased this recombinant protein from a company and treated wild-type mice after infection. Our observations demonstrated that although the results of the in vitro assay (Supplementary Materials Figure S3A–C) and body weight changes (Supplementary Materials Figure S3D) were negative, supplying recombinant VSIG4 protein conferred slight protection from influenza virus infections (Supplementary Materials Figure S3E). This suggests that recombinant VSIG4, or agents capable of enhancing its expression, may serve as promising therapeutic strategies for controlling inflammation, particularly during acute viral infections. Although this anti-inflammatory effect is evident, the precise mechanisms underlying VSIG4-mediated regulation remain to be fully elucidated. Notably, the clinical use of anti-inflammatory treatments based on recombinant proteins is not without precedent. Our study uncovers a previously underappreciated mechanism of protection in the context of influenza infection—namely, the role of immune homeostasis in conferring resistance to viral challenge. Together with existing clinical and experimental evidence, our results underscore the potential of adoptive cell therapies and immune-modulating strategies to enhance public health responses during outbreaks of emerging or severe viral infections.

## 5. Conclusions

Recent studies have revealed a complex interplay among inflammation, immunity, and infection. Our findings build upon this understanding by demonstrating that VSIG4 plays a critical role in maintaining immune homeostasis during acute viral infection. The growing body of evidence supporting the protective functions of VSIG4 across various infectious contexts not only enhances our understanding of its underlying mechanisms but also highlights the importance of considering immune homeostasis in the development of therapies for severe infections.

## Data Availability

The datasets generated during the current study are available from the correspondence author upon request.

## Supplementary Materials

The following supporting information can be downloaded at https://www.mdpi.com/article/10.3390/vaccines13101053/s1, Figure S1: Wild-type mice and VSIG4-deficient mice were monitored for survival, and their blood was harvested on day 15; Figure S2: VSIG4 gene deficiency upregulated expression of inflammatory factors in serum; Figure S3: Macrophages (RAW264.7) were treated with VISG4-Fc protein, and then infected with influenza PR8 (MOI = 0.01).
