# Supplementary material for: B7 Family Molecule VSIG4 Regulates Pulmonary Anti-Influenza Immune Responses via C-Type Lectin Signal Pathway
Source: Vaccines (Basel). 2025 Oct 14;13(10):1053. doi: 10.3390/vaccines13101053 (PMC12567591; doi:10.3390/vaccines13101053)
Supplement: Supplementary file 1 [file vaccines-13-01053-s001.zip › vaccines-3693132-supplementary.pdf]

Supplementary Figures

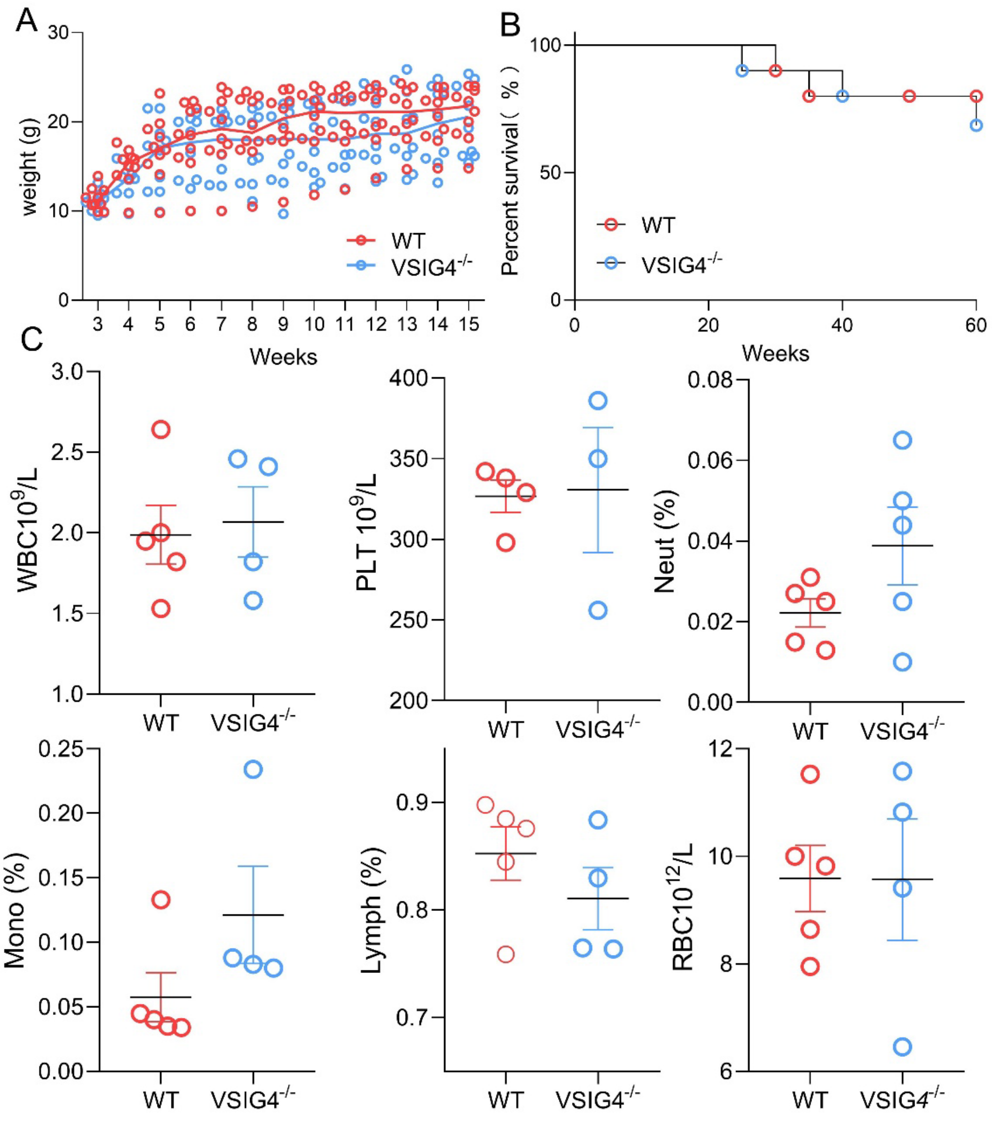

Supplementary Figure S1. Wild-type mice and VSIG4-deficient mice were monitored for survival, and their blood was harvested on day 15. (A), body weight; (B), survival rate; (C), blood parameters. All error bars are SEM.

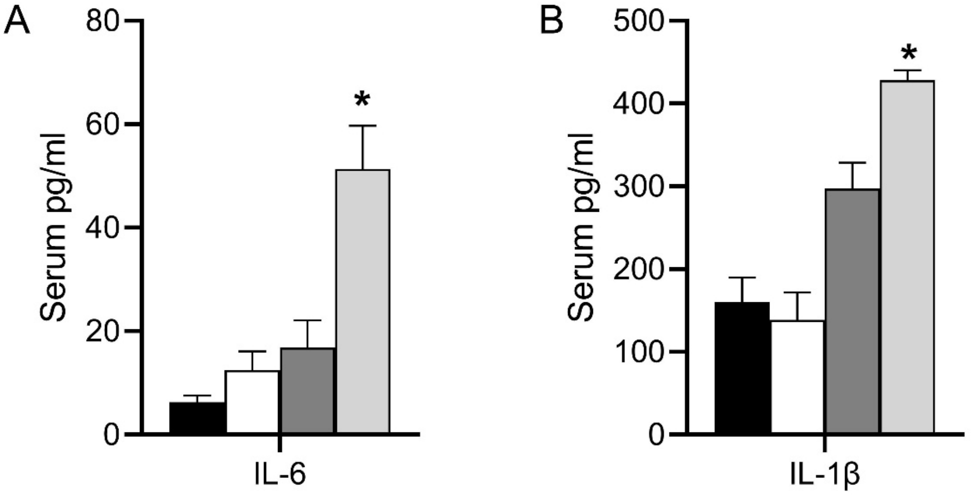

Supplementary Figure S2. VSIG4 gene deficiency upregulated expression of inflammatory factors in serum. VSIG4 gene-deficient mice and control mice were inoculated with a sublethal dose of influenza PR8 and their lung tissue was harvested on day 8. Serum levels of IL-6 (A) and IL-1β (B) were measured (n=8-10 per group). All data are shown with real concentration, but normalized with log 10 for a one-way ANOVA statistical test. All error bars are SEM; *p<0.05.

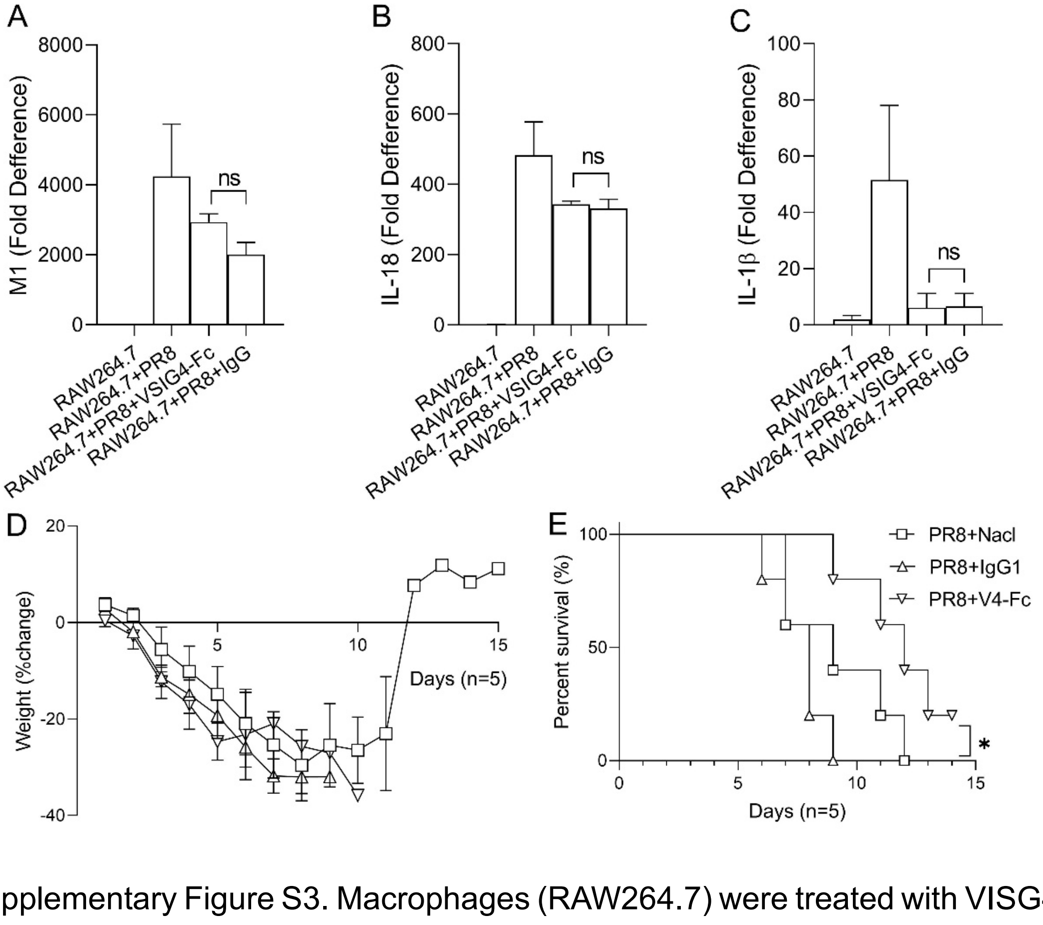

Supplementary Figure S3. Macrophages (RAW264.7) were treated with VISG4-

Fc protein, and then infected with influenza PR8 (MOI=0.01). The cell lysate was collected. The levels of viral load (A), IL-18 (B), and IL-1β (C) were measured. VSIG4 gene-deficient mice and control mice were infected with a sublethal dose of influenza PR8 and inoculated with 10 μg recombinant VSIG4 protein. The body weights (D) and survival (E) were documented. All error bars are SEM; *$p<0.05$.
